# Analytics for Investigation of Disease Outbreaks: Web-Based Analytics Facilitating Situational Awareness in Unfolding Disease Outbreaks

**DOI:** 10.2196/12032

**Published:** 2019-02-25

**Authors:** Nileena Velappan, Ashlynn Rae Daughton, Geoffrey Fairchild, William Earl Rosenberger, Nicholas Generous, Maneesha Elizabeth Chitanvis, Forest Michael Altherr, Lauren A Castro, Reid Priedhorsky, Esteban Luis Abeyta, Leslie A Naranjo, Attelia Dawn Hollander, Grace Vuyisich, Antonietta Maria Lillo, Emily Kathryn Cloyd, Ashvini Rajendra Vaidya, Alina Deshpande

**Affiliations:** 1 Los Alamos National Laboratory Los Alamos, NM United States; 2 Specifica Inc New Mexico Consortium Biological Laboratory Los Alamos, NM United States; 3 University of Virginia Charlottesville, VA United States

**Keywords:** epidemiology, infectious diseases, algorithm, public health informatics, web browser

## Abstract

**Background:**

Information from historical infectious disease outbreaks provides real-world data about outbreaks and their impacts on affected populations. These data can be used to develop a picture of an unfolding outbreak in its early stages, when incoming information is sparse and isolated, to identify effective control measures and guide their implementation.

**Objective:**

This study aimed to develop a publicly accessible Web-based visual analytic called Analytics for the Investigation of Disease Outbreaks (AIDO) that uses historical disease outbreak information for decision support and situational awareness of an unfolding outbreak.

**Methods:**

We developed an algorithm to allow the matching of unfolding outbreak data to a representative library of historical outbreaks. This process provides epidemiological clues that facilitate a user’s understanding of an unfolding outbreak and facilitates informed decisions about mitigation actions. Disease-specific properties to build a complete picture of the unfolding event were identified through a data-driven approach. A method of analogs approach was used to develop a short-term forecasting feature in the analytic. The 4 major steps involved in developing this tool were (1) collection of historic outbreak data and preparation of the representative library, (2) development of AIDO algorithms, (3) development of user interface and associated visuals, and (4) verification and validation.

**Results:**

The tool currently includes representative historical outbreaks for 39 infectious diseases with over 600 diverse outbreaks. We identified 27 different properties categorized into 3 broad domains (population, location, and disease) that were used to evaluate outbreaks across all diseases for their effect on case count and duration of an outbreak. Statistical analyses revealed disease-specific properties from this set that were included in the disease-specific similarity algorithm. Although there were some similarities across diseases, we found that statistically important properties tend to vary, even between similar diseases. This may be because of our emphasis on including diverse *representative* outbreak presentations in our libraries. AIDO algorithm evaluations (similarity algorithm and short-term forecasting) were conducted using 4 case studies and we have shown details for the Q fever outbreak in Bilbao, Spain (2014), using data from the early stages of the outbreak. Using data from only the initial 2 weeks, AIDO identified historical outbreaks that were very similar in terms of their epidemiological picture (case count, duration, source of exposure, and urban setting). The short-term forecasting algorithm accurately predicted case count and duration for the unfolding outbreak.

**Conclusions:**

AIDO is a decision support tool that facilitates increased situational awareness during an unfolding outbreak and enables informed decisions on mitigation strategies. AIDO analytics are available to epidemiologists across the globe with access to internet, at no cost. In this study, we presented a new approach to applying historical outbreak data to provide actionable information during the early stages of an unfolding infectious disease outbreak.

## Introduction

### Challenges in Outbreak Investigation

Infectious diseases continue to be a leading cause of mortality worldwide despite substantial advances in public health [1]. Disease outbreaks are not bound by national borders and can have far-reaching economic and social impacts. Therefore, early detection and monitoring are key to curtailing unfolding outbreaks. Tools and analytics that improve situational awareness can aid communication in the initial stages of an outbreak and enable effective decisions for outbreak control [2].

Traditionally, historical outbreak data have been used to enhance and develop disease forecasting models. For example, when modeling influenza, Viboud et al utilized the method of analogs, which uses weighted vectors of historical time series data that match current activity to build forecasts 1 to 10 weeks ahead [3]. Relatedly, Sugihara and May utilized a library of historical measles and chickenpox outbreaks to understand historical patterns in variation and develop short-term forecasting models [4]. In addition, extrapolation of retrospective data has proven useful in resource-poor areas, for example, to establish levels of antimicrobial resistance in areas with minimal surveillance [5]. More broadly, historical disease data are often used for model parameter estimation [6-8]. Data used are often not confined to epidemiological data. For example, climate data have been used to develop outbreak models that are affected by environmental factors, such as malaria, dengue, and cholera [9]. However, these data are typically difficult to use owing to poor organization and integration. Extracting relevant information from official reports is time consuming.

Epidemiological data are rarely easily accessible or available in an easily analyzed format. There are efforts to circumvent these issues, such as Project Tycho [10,11] and Gideon [12], but these Web applications have limitations. Project Tycho is currently limited to data from the National Notifiable Disease Surveillance System in the United States and Gideon offers its data for a fee, which may prove to be prohibitive for some public health users. Furthermore, neither provide information to contextualize or describe historical outbreaks nor the tools to meaningfully relate a present situation to a past one.

### Rapid, Facile Decision Support Using Historical Data

To the authors’ knowledge, there have been no previous formal attempts to build a decision-support application based on historical data. However, historical analogies have been utilized for disease projection and forecasting. In 2010, Haiti experienced its first cholera outbreak in over 100 years, months after being struck by an earthquake that damaged its infrastructure and gave rise to poor sanitary conditions [13]. In the days following the first case notifications, the US Centers for Disease Control and Prevention (CDC) contextualized the Haiti outbreak by comparing it with a similar outbreak in Peru [14]. As more surveillance data became available and forecasting models were developed, more complex projections were established. However, quick comparisons, such as the historical analogy model that provided a notification to the Ministere de la Sante Publique de la Population of Haiti of the need to prepare for a large epidemic [15].

Currently, internet access is widely available around the globe. More than 40% of the world’s population has access to the World Wide Web [16]. Hence, Web-based analytics that facilitate outbreak investigation have the potential to improve outbreak control around the world. In this study, we presented a Web-based visual analytic called Analytics for the Investigation of Disease Outbreaks (AIDO) available under the domain name bsvgateway.org [17], which has been developed to facilitate situational awareness in the early stages of an infectious disease outbreak. AIDO allows matching of unfolding outbreak data to a representative library of historical outbreaks and provides epidemiological clues that facilitate a user’s understanding of an unfolding outbreak and enables informed decisions on mitigation actions. This tool currently has analytics for 28 infectious diseases and contains a *browse library* for an additional 11 diseases. We described the methodology used to develop this tool and illustrated the utility of the tool using 4 case studies, one of which is described in detail. We offer AIDO as a rapid, easy-to-use no cost analytic for outbreak investigation and short-term forecasting.

## Methods

To develop AIDO, we used the following iterative process: (1) collect historical outbreak data; (2) develop AIDO algorithms; (3) develop the user interface, additional visuals, and functionalities; and (4) perform verification and validation. In this section, we have described the process for developing a disease-specific representative historical outbreak library, the associated algorithms, and the AIDO interface.

### Collect Historical Outbreak Data

AIDO contains representative outbreak data for 39 diseases (Multimedia Appendix 1). We defined a representative library as one that includes outbreaks with a broad range of cumulative case counts, outbreak durations, diverse circumstances, and which occur in a variety of locations. Outbreak data were identified using official academic and government data, as well as retrospective studies. Data sources included ProMED [18,19], CDC [20], World Health Organization [21,22], Eurosurveillance [23], European Centre for Disease Prevention and Control Disease Reports [24], and government Ministry of Health databases (available from Biosurveillance Resource Directory under the domain name bsvgateway.org) [25], as well as other scholarly journals available on PubMed and Google Scholar. If data were only available in the graphical form (eg, graphs in a PDF report or peer-reviewed publication), plots were digitized using PlotDigitizer [26].

To be considered for inclusion in a disease library, outbreak data must contain (1) a time series of case counts, (2) enough associated data to perform property analysis (described below), and (3) enough metadata to annotate the outbreak (described below). To apply analytics to a disease library, there must be (1) a minimum of 10 outbreaks included per disease library and (2) sufficient data to complete the property identification protocol (described below). Our analytics rely on a robust library; therefore, it is necessary to have a minimum threshold. A total of 10 outbreaks were considered to be a reasonable lower limit and produced reasonable results. In general, outbreaks that occurred during or after the year 2000 were prioritized for inclusion to represent current natural and built environments. However, in cases of rare diseases, outbreaks from previous years were included to achieve the minimum threshold for analysis (eg, both Ebola and Marburg libraries include outbreaks that occurred before 2000).

In addition to the outbreak time series, detailed information on factors that influenced the outbreak was also collected and used to describe each outbreak. Information collected included index case, important dates, the vector (if applicable), transmission routes, pathogen classification, case definition, geographic and historical information, and identified risk factors and control measures that were implemented.

### Algorithm Development

#### Similarity Algorithm

The similarity algorithm identifies outbreaks similar to the user’s unfolding situation through a similarity score that is computed as a sum of values assigned to weighted properties specific to a disease. The algorithm has 3 components: (1) disease-specific properties; (2) weights calculated for each property; and (3) computation of the weighted sum by the AIDO algorithm. Here, we have described the statistical process used to identify properties, the procedure used to weight properties for relative importance, and the final equation used in this algorithm. Diseases with less than 10 outbreaks do not have an associated similarity algorithm and are represented in AIDO as *browse-only*.

Property selection: In AIDO, properties are characteristics that influence the case count or duration of outbreaks. In essence, these are the population-level signatures that help match a user’s situation to outbreaks in our library. There were 3 types of properties: (1) categorical (eg, vaccination status: 90% to 100%, 80% to 89%, 50% to 79%, and less than 50%), (2) continuous (eg, physician density: range of values 0.1 to 10), and (3) binary (eg, population movement: yes or no). Properties were discretized if extant literature supported categorization of continuous variables. We identified 27 different properties that were used to evaluate outbreaks across all diseases for their effect on case count and duration of an outbreak. These properties and their definitions are included in Table 1. Properties were further categorized into 3 domains.

**Table 1:** Properties collected for statistical analysis.

Name (variable type)		Description
**Domain: population**		
	Population (continuous)	Population of affected location as a continuous variable
	Population (categorical)	Population of affected location. Discretized into groups based on orders of magnitude
	Disease status (binary)	Endemicity status (ie, endemic or nonendemic to the region) during the time of the outbreak
	Rural versus urban (binary)	Binary categorization of the relative population density of the outbreak’s location
	Age stratification (categorical)	Relevant age categories or median age of reported cases. (Varies by disease; groupings are identified using published literature)
	Special population group (binary)	Binary (yes or no) indicator describing if the outbreak occurred in the general population or a particular group with a specific common exposure or risk factor
	Vaccination status (categorical)	Vaccine coverage (%) of the country and/or region of interest
	Population movement (binary)	Indication of whether or not large-scale population movement (eg, mass migration and influx of a refugee population) was an influential component of the outbreak
	Sex (continuous)	Fraction of cases in males (identified using the literature)
**Domain: location**		
	Climate (categorical)	Climate type corresponding to the location of interest, represented as the first letter of the Köppen-Geiger climate classification key (A, B, C, D, and E) [27,28]
	Season (categorical)	Time of year (Spring, Summer, Autumn, and Winter) during which the majority of the outbreak occurred
	Precipitation (categorical)	Precipitation category corresponding to the location of interest, represented as the second letter in the Köppen-Geiger climate classification (f, m, w, s, W, S, T, and F)
	Rainy versus dry (binary)	Binary indicator describing the typical weather patterns (ie, rainy or dry) in the location at the start of the outbreak
	Natural disaster (binary)	Binary indicator describing if a natural disaster appeared to be associated with the onset of the outbreak
	Human Development Index (HDI; categorical or continuous)	HDI in the location and year of interest [29]. Both categorical and continuous values were tested. In the event that both properties were significantly related to outcomes, categorical values were preferred because of they are more usable within the user interface.
	Physician density (continuous)	Physician density per 1000 persons in the year of interest, or the most recent year reported [30]
**Domain: disease**		
	Pathogen source (categorical)	Main source of exposure to the pathogen of interest
	Outbreak curve (categorical)	Type of outbreak as reflected in the outbreak curve shape (point source, common source, and propagated outbreak)
	Vector type (categorical)	The most relevant genus/species/classification of the disease vector
	Case fatality rate (CFR; continuous)	Percent of fatal cases
	Attack rate (continuous)	Number of new cases per 1000 persons
	Case definition (categorical)	Classification of reported cases (suspected, probable, confirmed, or any combination thereof)
	Disease presentation classification (categorical)	Description of clinical disease presentation (eg, bubonic plague and pneumonic plague)
	Animal contact (binary or categorical)	Reported contact with potentially infectious animal (used for zoonotic diseases). Can be binary (yes or no) or categorical (ie, contact with particular animal), depending on the level of data available in literature
	Contamination source (categorical)	Product or site epidemiologically linked to the outbreak (used for foodborne illnesses)
	Transmission mode (categorical)	Mode of transmission that best characterizes the majority of disease spread during the outbreak (eg, airborne and direct contact)
	Outbreak source proximity (categorical)	Geographic proximity of cases to a known or likely source of contamination
	Outbreak pathogen (categorical)	Etiological agent

Available data for all properties were collected for each outbreak in AIDO. Statistical analyses were used to identify which properties separated outbreaks on the basis of outbreak size or duration for use in the similarity algorithm described in the following equation.

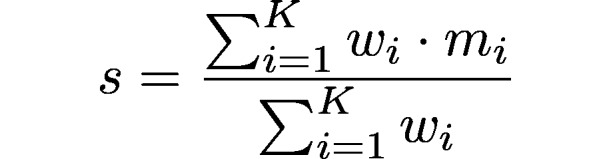



Outbreak similarity scores are generated using this simple weighted sum such that 0≤*w_i_*, *m_i_* ≤1 and the sum of all *w_i_*=1, which ensure that 0≤*s* ≤1. Here, *s* is the outbreak’s similarity score, *K* is the number of parameters considered, *w_i_* is the weight of parameter *i*, and *m_i_* is the outbreak’s match score of parameter *i* (ie, how well the outbreak’s value for parameter *i* matches the user’s value provided in the query form). Note that although this equation returns a score, *s*, between 0 and 1, AIDO displays scores as percentages (ie, *s* ⋅100).

Statistical analyses were conducted to determine which properties to include in the disease specific similarity algorithm based on relationships with outbreak magnitude and duration. A conclusion that there is a meaningful association (ie, statistical significance or a strong enough correlation) indicates that this property helps to distinguish outbreaks of different magnitude or length from one another, and thus can be used to find outbreaks most similar to a user’s data. The results from the property analysis do not reflect or intend to imply any causative relationship between a property and outbreaks of a given disease. A statistical association is merely reflective of a property’s relative ability to discriminate between outbreaks of different sizes and lengths.

**Figure 1:**
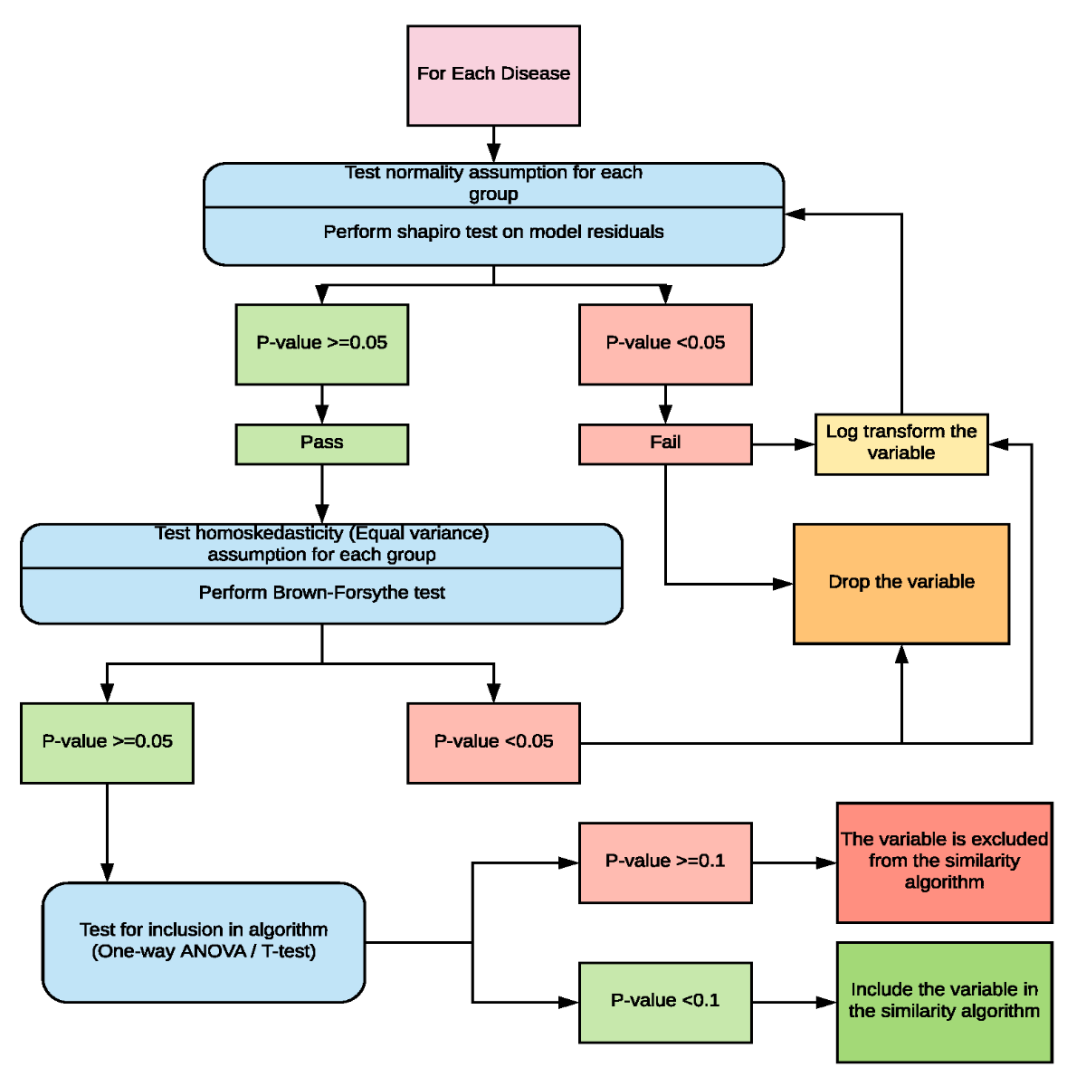
Schematic depicting the flow of statistical operations performed on categorical properties during property analysis.

To perform the statistical analysis, a series of statistical tests were automated using R (R-foundation) [31]. Properties were segregated into their variable type: binary, categorical (multilevel), or continuous. Figure 1 illustrates the process by which these statistical analyses were completed for categorical variables. For a given disease, after identifying all properties listed in Table 1, statistical assessments first measured whether or not the data met the assumptions of normality for the distribution of residuals as well as equality of variance (homoscedasticity) by performing the Shapiro-Wilk and Brown-Forsythe tests, respectively. For continuous variables, only the distribution of the residuals was assessed, and for instances where the distribution was normal (Shapiro-Wilk test), a Pearson correlation was run. A Spearman correlation was performed on properties with non-normal distributions. We assume that all values for dependent property variables (both case count and duration) are independent, as we did not have any prior knowledge that these values are dependent on one another in any way. As our data are curated from available scientific literature and published official reports from different locations across different years, we assume all individual values to be independent from other values and outbreaks in our library.

Following assessments of normality and homoscedasticity, statistical tests of association, depending on variable type, were performed (either T-test, one-way analysis of variance (ANOVA), or Pearson or Spearman correlation) to assess each property’s relationship with both case count and duration, independently. *t* test and ANOVA *F* test results were assessed at a significance level of 0.05, whereas a correlation above 0.30 was considered a meaningful association [32]. Properties that did not meet these criteria were excluded from the similarity algorithm, whereas those that did meet these criteria were included.

Weight calculation for selected properties: In the previous sections, analyses of outbreak data have been dedicated to determining which properties correlate most strongly with the duration and number of cases in the outbreaks of a given disease. The ultimate goal of these properties is to allow AIDO to sort the historical outbreaks based on the user’s input. The exact process used to create this ranking is described in later sections, but in short, the similarity algorithm is a function that maps the case count, duration, and disease-specific properties of the user’s input and the historical outbreak to a number between 0 and 1. The set of values that are used by the similarity score are referred to as *parameters*. These include the user’s input for case count and duration.

After determining which parameters would be included in the algorithm, a modified sensitivity analysis was used to determine the weights for each parameter. The sensitivity analysis determines the relative importance of selected parameters to size and duration of the outbreak. For all algorithms, the first 2 parameters are case count and duration, which were given the greatest weight and were not considered in the processing done for the weighting algorithm. For the additional disease-specific properties, weights were calculated using an automatic algorithm that compares each outbreak to all other outbreaks for a given disease. The effect of each property on the ranking of outbreaks in the library was evaluated. If removing a property had a large effect on the order of the outbreaks, it was inferred that the excluded property was important and should be given a greater weight. This evaluation was conducted for all properties for a given disease, and relative ranking or weights were determined. The sum of all weights was set to 1. A more detailed description of the process is given in Multimedia Appendix 2.

#### Additional Analytics

In addition to the similarity algorithm, AIDO includes a number of other visual analytics designed to enhance situational awareness. Use of these analytics is illustrated in the Results section, Case Study, and associated figures.

Short-term forecasting: The AIDO disease library can also be used to perform short-term forecasting using the method of analogs (similar to, but simplified from, the study of Viboud et al described above [3]) approach. As a first step in this method, the cumulative case count curves for all outbreaks for the disease of interest were aligned in time. The distance criterion used in AIDO is simpler than that used in Viboud et al; in AIDO, because outbreaks are first deliberately aligned in time and because AIDO stores *representative* outbreaks and not *all* outbreaks, all available outbreaks are used in the analysis (ie, without using all outbreaks, there would not be enough data to attempt the analysis). The mean and SD at each time point were calculated and fit to a normal distribution. This distribution was then used to find the median, 50% prediction interval (PI) and 90% PI at each time unit. AIDO institutes a lower bound of zero (case counts cannot be below zero). To customize the forecast to the user, each case count is weighted in proportion to its outbreak similarity score; thus, case counts in outbreaks that are scored higher weigh more than case counts in outbreaks that are scored lower. To achieve this, AIDO computed weighted mean and SD values, which were then used as the normal distribution’s parameters.

AIDO currently requires at least 10 data points at each time point. Once there are fewer than 10 data points, the forecast stops. If necessary, AIDO will use cubic B-spline interpolation to handle time series interval granularity issues. For example, if there are both monthly and weekly data, cubic B-spline interpolation will be used to fill in the gaps in the monthly data so that they can be used alongside the weekly data. AIDO interpolates at the finest resolution present in each outbreak library; although interpolating epidemiological data may introduce errors into the forecasting algorithm, the authors felt that because the method of analogs is already a rough forecasting measure that in practice yields reasonably large PIs, the additional error provided by interpolation would be unsubstantial.

The method of analogs approach has been applied in fields such as meteorology, climatology, and epidemiology [3,33,34]. When a user sorts results by similarity score, a short-term forecast is displayed at the top of the sorted outbreak results. This graph presents a simple custom forecast of cumulative disease incidence based on user input and our library of historical outbreak curves.

Anomaly detection: AIDO features an anomaly detection component that enables a user to compare their values to the values in AIDO’s historical outbreak database. Rather than employing a specific anomaly detection algorithm, AIDO offers a simple visual approach to guide the user’s analysis and identify anomalies. We display the user’s value among all outbreak values so that the user can visually see where their values lie. For example, for discrete variables, we draw a pie chart and highlight the section that the user falls into; if the user’s slice represents only 5% of all outbreaks, for example, then this may be indicative of an anomaly. For continuous variables, we plot all outbreak points and show the corresponding box plot and highlight the user’s value; here, an anomaly may be if the user’s point lies within the outliers. An anomaly for the user’s data is easily seen through such visualizations and directs the user’s attention to key features of their unfolding event that may warrant further investigation.

Owing to data sparsity issues and significant differences between disease presentations, AIDO focuses on a qualitative, rather than quantitative, anomaly detection approach that requires user engagement and interpretation. Future work, however, could include automated quantitative anomaly detection results. There are a number of unsupervised anomaly detection algorithms that could be explored [35]. For example, classifier-adjusted density estimation (CADE) is a promising nonparametric unsupervised anomaly detection algorithm [36,37]; CADE and many other such algorithms, however, often require a significant volume of data for analysis, which can prove to be difficult when epidemiological data are used.

#### Developing the User Interface, Additional Visuals, and Functionalities

AIDO functionalities are written in Python [38] using the Django [39] Web framework and PostgreSQL [40] for the backend. Bootstrap [41], jQuery [42], and Plotly [43] are used on the front end for overall user interface design or functionality and graphs. The AIDO homepage and various features of the user interface are described in the Results section.

Outbreak comparisons, browsing and related outbreaks: An outbreak comparison graph is displayed on each result page that shows the point estimate for user’s values in comparison to the 5 outbreaks listed on that page. In addition to the analytics provided, the interface allows users to explore the outbreaks available in each library without making use of the analytic components. We refer to this as *browsing* the library. Utility of these features were evaluated in the case study detailed in the Results section.

We noted in our analysis of representative libraries that some outbreaks in our libraries have meaningful relationships between each other. For example, there are some instances of outbreaks that were related because of common exposure to contaminated food that is spread to multiple locations in a country. Other times, outbreaks might be related by virtue of an individual seeding a new outbreak of the same illness in a new location. AIDO provides such information to the user if this option is selected in the analytic.

#### Performing Verification and Validation

Verification of AIDO was performed using more than 200 automated tests. These tests are run automatically every time the code base is changed, allowing an alarm to be raised before deployment if an error in the codebase is detected. In addition, we performed two manual tests after implementation of disease-specific algorithms in AIDO. The first uses data for outbreaks already in the library to verify a 100% similarity match between identical outbreaks. The second uses outbreaks that were not included in the library as test case scenarios. Here, we qualitatively observe if the highest matching outbreaks are similar to the situation from the test outbreak.

AIDO also underwent extensive user interface evaluation and user experience testing by several external entities such as Massachusetts Institute of Technology Lincoln Labs, a user interface design class from the University of Washington, the Fusion Division within the Office of the Assistant Secretary for Preparedness and Response in the US Department of Health Human Services, Science and Technology Directorate in the US Department of Homeland Security, community health epidemiologists in the state of New Mexico, US CDC, and the National Bio-surveillance Integration Center.

## Results

### Disease Libraries and Algorithm Properties

**Figure 2:**
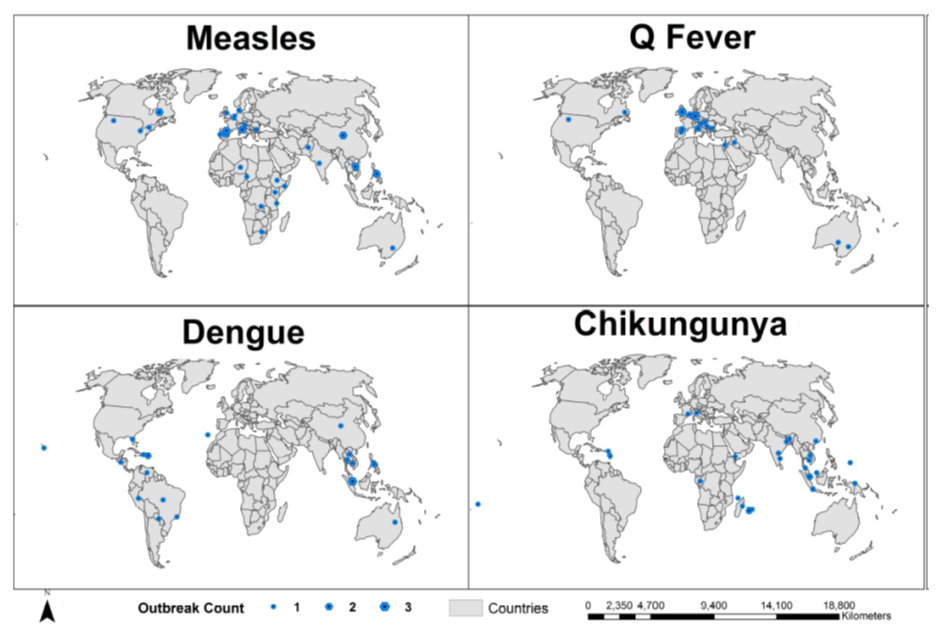
Geographic spread of historical libraries for four diseases. Points are proportional in size to the number of outbreaks in that country within our library.

Currently, AIDO contains 673 outbreaks across 39 different diseases. Figure 2 illustrates the geographic breadth and multicontinent coverage of outbreaks for 4 diseases (measles, Q fever, dengue, and chikungunya). Multimedia Appendix 1 provides data on the total number of outbreaks, geographical distribution, and algorithm properties for all diseases in AIDO. The properties identified for the similarity algorithms for the 4 diseases are shown in Table 2. Unsurprisingly, precipitation and climate were identified as relevant properties for mosquito-borne diseases such as chikungunya and dengue. Vaccination coverage was found to be important for measles outbreaks and animal-specific properties were considered important for Q fever (a zoonotic disease).

**Table 2:** Measles, Q fever, dengue, and chikungunya algorithm properties.

Disease	Algorithm Properties
Measles	Vaccination status (country)
	Vaccination status (region)
	Physician density
	Climate
Q fever	Animal contact
	HDI^a^
	Affected animal
	Outbreak source proximity
Dengue	Physician density
	Climate
	Population (discrete)
Chikungunya	Precipitation
	HDI

^a^HDI: Human Development Index.

Statistically significant algorithm properties for the 4 diseases highlighted in the study are given.

An analysis of properties identified for diseases with similar characteristics was conducted to identify trends in properties across similar diseases Multimedia Appendix 3 show the comparison of properties for mosquito-borne diseases and vaccine preventable diseases that are part of the AIDO library. Although there are some similarities across diseases, we find that statistically important properties tend to vary, even between similar diseases, which one may not expect given common modes of transmission such as mosquitoes as vectors or vaccine-preventable diseases. This may be due to our emphasis on including diverse *representative* outbreak presentations in our libraries.

### Case Study: Q Fever Outbreak in Bilbao, Spain 2014

To describe a plausible use case in AIDO, we present a case study on a Q fever outbreak in Bilbao, Spain, in 2014. This outbreak was described in depth by Alonso et al [44]. This disease and outbreak combination was selected because available data were detailed enough to illustrate multiple features of AIDO.

**Figure 3:**
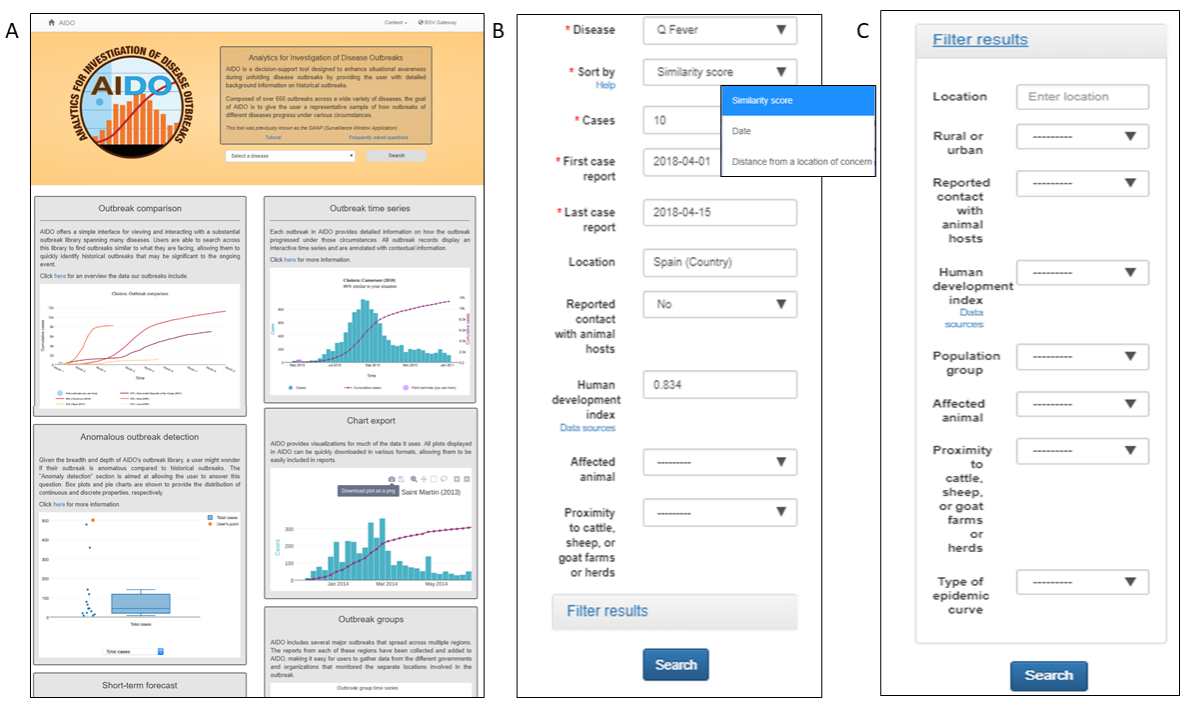
AIDO data input forms. Panel A shows the AIDO home page and a drop down menu with Q-fever selected. This page also contains links to a tutorial, frequently asked questions, and a feedback form. Panel B shows the user input form, filled with data for the Bilbao outbreak. Panel C shows the filter options available for analysis.

AIDO user input data: Q fever was selected as the disease of interest from the AIDO homepage and the following information was used to populate the user input form (Figure 3). This outbreak occurred among workers at a waste-sorting plant in Bilbao, Spain, between February and April 2014. Approximately 10 cases were reported in the first 2 weeks of the outbreak [44]. The plant employed about 100 employees, was located in a municipality, and the patients did not report regular contact with animal hosts [44]. The human development index (HDI) for Spain in 2014 was 0.834 [28]. Information about animal hosts and proximity to the farm was not available during the early stages of this outbreak and was left blank. Figure 3 also shows the ability to sort the AIDO library for Q fever based on date and distance from location and illustrates the expanded *filter results* that can be used to narrow down outbreaks with specific properties.

**Figure 4:**
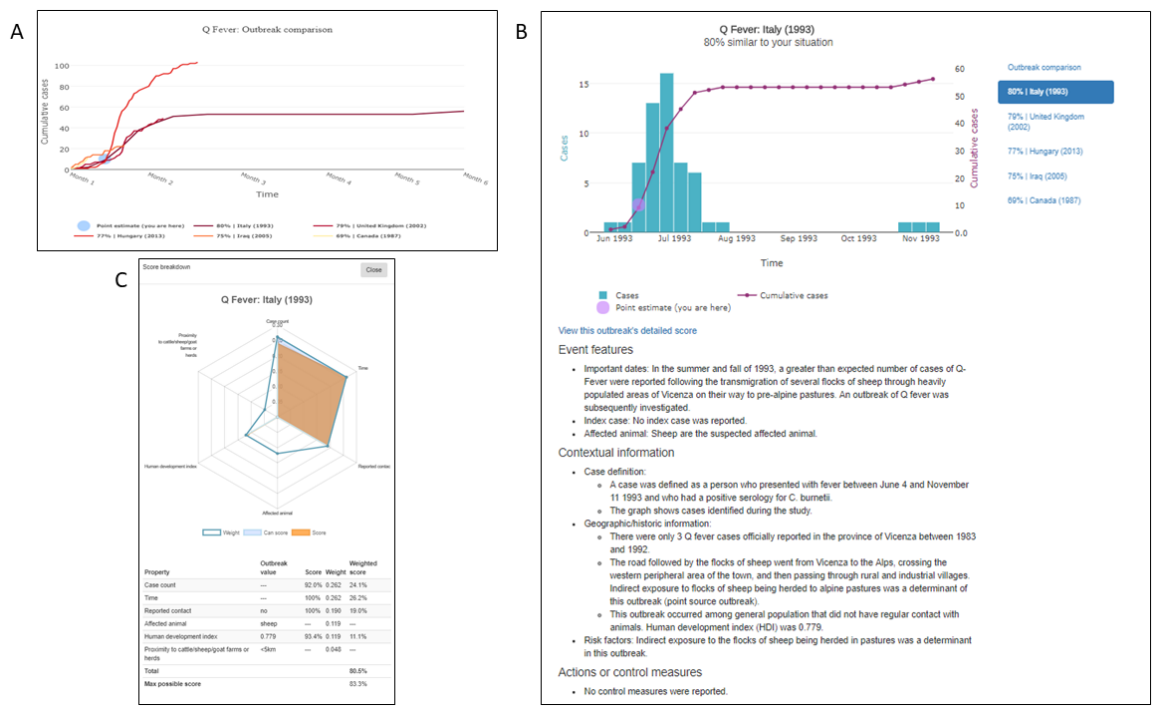
AIDO case study: Q-fever outbreak in in Bilbao, Spain in 2014. Panel A shows the outbreak comparison graph for the five most similar outbreaks, and a point estimate reflecting the user's situation in this context. Here, line colors with higher saturation correspond to higher similarity. In panel B, the graph shows an outbreak time series for a Q Fever outbreak in Italy in 1993. Panel C shows a breakdown of the similarity score between the user's unfolding outbreak and the historical outbreak. All graphs presented in AIDO are interactive and available for download in multiple formats.

Analysis of AIDO output: After completion of the similarity algorithm computation, the AIDO output showed the top matching outbreaks in the outbreak comparison graph and details of the five most similar outbreaks on page 1 (Figure 4). An example of information provided for each outbreak in AIDO is given here. In this case study, the outbreak comparison graph showed that the 5 most similar outbreaks had cumulative cases ranging from 10 to 100 cases and a total duration of 2 weeks to 6 months. The Q fever outbreak reported from Italy in 1993 and United Kingdom (2000) were the most similar outbreaks (80% and 79% similarity, respectively; Figure 4 shows the Italian outbreak). A radar plot is used to illustrate computation of the similarity score. This plot can be accessed through the *view this outbreak’s detailed score* hyperlink under the epidemic curve graph. Further examination of the metadata showed that both outbreaks were caused by environmental exposure (sheep migration and contaminated strawboard in a factory office, respectively). This is similar to the probable cause of the outbreak in Bilbao, animal remains that had contaminated the waste-sorting environment at a factory [44].

**Figure 5:**
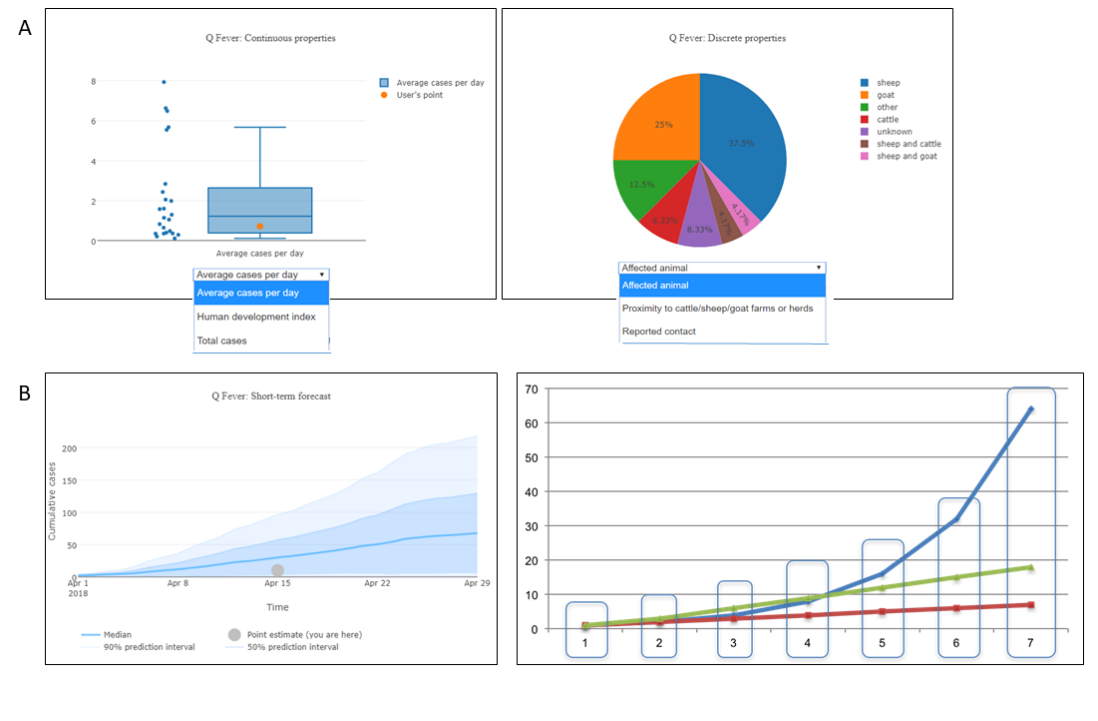
Additional analytics-anomaly detection and short-term forecast. Panel A illustrates two types of graphs used in the anomaly detection tab. Continuous variables (e.g., average cases per day, population at risk, or total cases) are shown as box plots. Discrete or categorical variables (e.g., season or affected animal) are shown as pie charts. The example presented shows that the case study outbreak is similar to other outbreaks included in our library. Panel B shows short term forecasting using the method of analogs. The data shown here can be used to estimate cumulative case count. This figure also demonstrates how data points are aligned for the short-term forecast.

**Figure 6:**
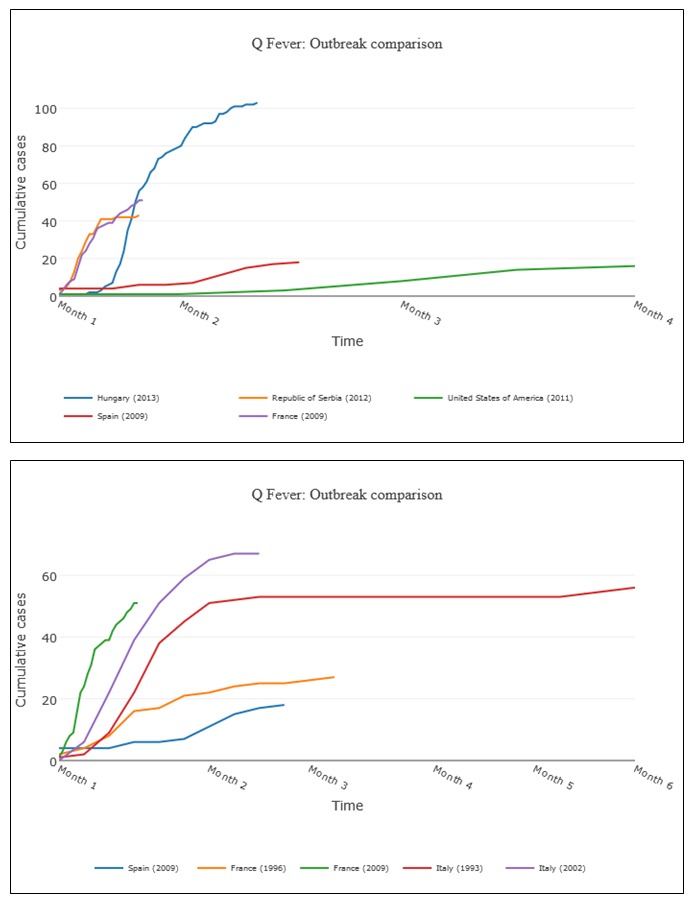
Browse functionality. This figure demonstrates browse functionality by date and by location available on AIDO.

The other 3 outbreaks with the highest similarity score included outbreaks from Hungary in 2013 (77%), Iraq in 2005 (75%), and Canada in 1987 (69%). The outbreak factors revealed that these epidemics also occurred among small groups of people associated by work and that the case count and duration were similar. Analysis using the anomaly detection features (Figure 5 top panel) showed that the case study outbreak parameters fell within the normal range for natural outbreaks and revealed high likelihood of the waste materials of sheep and goats as the cause of this outbreak. The short-term forecasting algorithm predicted a mean cumulative case count of 50 to 100 cases (Figure 5 bottom panel). Figure 6 shows AIDO outbreaks sorted by date and distance from the location (Spain).

Using AIDO’s analysis, it could be hypothesized that the case study outbreak in Spain was likely to have 30 to 50 cases during the initial one to two months of the outbreak. This was confirmed by Alonso et al, who showed that the Spanish outbreak reported 45 cases from February 17 to April 27, 2014 [44]. The short-term forecast analysis in AIDO accurately predicted the expected case count and duration for the unfolding outbreak using only data from the initial 2 weeks. This case study shows that AIDO can be used during initial stages of an outbreak with minimum input data and information on expected case count and duration, and possible causes can be gleaned from the analysis. AIDO analysis can be performed iteratively as the outbreak progresses.

**Figure 7:**
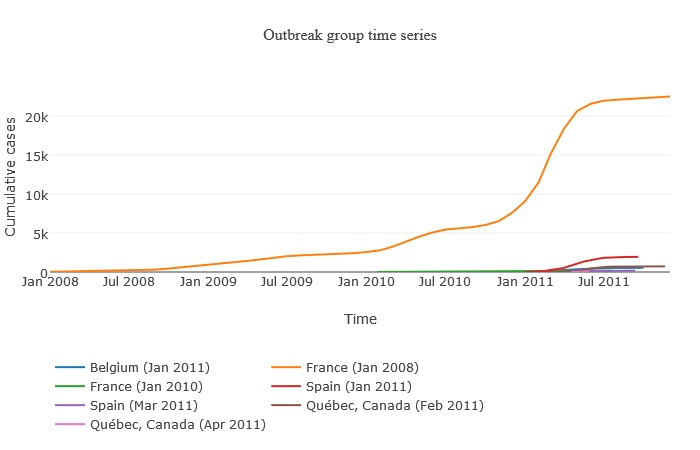
Example of the related outbreak interface. Here, we show outbreaks related to the France 2008-2011 measles epidemic. All graphs presented in AIDO are interactive and available for download in multiple formats.

The AIDO Q fever library did not contain any related outbreaks. Figure 7 describes the related outbreak feature by showing the various outbreaks connected to the France 2008-2011 measles outbreak. These graphs provide information on the timeline associated with the start of related outbreaks and alerts the user on the possibility of the unfolding outbreak being part of a larger phenomenon. The *view related outbreaks* hyperlink under relevant outbreaks provides access to the related outbreak information.

All disease libraries in AIDO were evaluated using multiple case studies similar to the Q fever case study presented above. Details on 3 additional case studies for chikungunya [45], measles [46], and dengue [47-49] are given in Multimedia Appendix 4. These case studies also demonstrate the utility of AIDO analysis during the unfolding stage (3 to 4 weeks) of an outbreak to identify possible case count, duration, and distinctive features during the epidemic.

## Discussion

AIDO presents relevant outbreak data in a visually concise manner with graphs and point estimates of the user’s input scenario in the context of the historical outbreak. Its analysis can provide an estimated case count and duration (short-term forecasting) and outbreak control measures that were effective in the past and AIDO facilitates delivery of outbreak information in an easy-to-interpret format.

AIDO is intended for members of the infectious disease surveillance community, both at the global level as well as the local level. A nongovernmental organization studying an ongoing outbreak can use this tool to analyze the scope of a current outbreak in any part of the world and devise control strategies that have proven effective in similar historical outbreaks. An individual physician may find this tool useful in understanding their case counts in a wider context and help facilitate their decision-making process on reporting relevant data to authorities. Individual analysts or local epidemiologists can use this tool as an aid in accessing the ongoing outbreak with increased situational awareness on what happens in their region and in similar regions around the world. In effect, this feature provides a projection of how the outbreak may unfold and could be considered a form of forecasting.

The algorithms and visuals available in AIDO inform users about the historical and geographical context of outbreaks for a given disease. The analysis also increases a user’s knowledge on diverse outbreak scenarios associated with a given disease. This information may enhance understanding on possible routes for outbreak progression (eg, transportation-associated global transmission). The related outbreak feature of AIDO can be utilized to generate hypotheses on next *hot spots* for a given outbreak and improve surveillance efforts in those locations. The anomaly detection algorithm on AIDO was specifically designed to detect anomalous presentations of an unfolding outbreak, perhaps biothreat scenarios. External evaluators of AIDO described it as *research at fingertips* for analysts or users. AIDO can also be used in education or training of epidemiologists.

Historical data have been used to develop epidemiological models. However, most models have challenges becoming operationalized owing to a variety of reasons [50]. AIDO is a tool that combines extensive epidemiological data with novel but simple analytics to contextualize and generate hypotheses about the trajectory of unfolding outbreaks. Rather than focusing on complex epidemiological models (which we recognize have substantial use and utility in the field), we take a historical approach and aim to identify relevant events that have already happened. We believe that this approach is novel and can provide complementary information to that derived from traditional modeling approaches.

### Limitations

It is important to note that our approach relies on a few recognized limitations. First, we know from prior research that historical data are subject to change and are not fully complete [51]. To minimize this known bias, we used the most complete data available. However, it is likely that the data presented in our libraries include some known reporting bias.

Relatedly, the outbreak-matching algorithm depends on the diversity of historical outbreaks that exist in the library. This may particularly affect AIDO when investigating outbreaks with no precedence. For example, the 2014 Ebola outbreak in West Africa had no historical counterparts. Using AIDO in these situations may prove to be less reliable. However, we also believe that the anomaly detection functions provide use in these unprecedented situations.

As our libraries are created using publicly available data, it is also likely that our libraries are slightly skewed toward large or highly publicized outbreaks. Furthermore, because of this bias, data are much more prevalent in nations with robust surveillance systems. For example, Chase-Topping et al describe this trend with respect to worldwide incidence of *Escherichia coli* O157. Although food contamination of *E. coli* is present worldwide, outbreak investigations are conducted almost exclusively by developed nations [52]. It is because of this known bias that we aim for representative outbreak libraries that showcase the known breadth of disease outbreak presentation. However, it is unlikely that our effort to create representative libraries completely eliminates this bias.

AIDO data are largely limited to diseases affecting humans. Although we have libraries for Porcine Epidemic Diarrhea Virus and Foot and Mouth Disease, attempts to expand to other animal diseases have been challenging. This is simply due to a lack of data; time series data are difficult to obtain for animal diseases and almost nonexistent in plant outbreaks. These types of data are economically sensitive and, as a result, are not often reported.

Additionally, our short-term forecasting component relies on a method of analogs approach that assumes normally distributed data, which may not always be a good assumption. As a result, in some cases, the short-term forecast may not be reliable.

Finally, we wish to draw attention to some important considerations of our statistical analysis for property identification. As our procedure specifically identifies properties that are statistically related to outbreak size or duration, any updates to the disease library can, by design, change the related properties. Therefore, the property analysis must be performed any time there is a change in data. Automation of these processes is planned as a future project.

### Future Work

Data in AIDO are constantly being updated. Much of this work is performed manually by a team of biologists and public health experts. However, we have also created the infrastructure to crowdsource data. AIDO includes a feedback form that allows the user to send us information about an outbreak currently not included in the library. The popularization of these types of decision support tools and inclusion of outbreak data from developing nations will facilitate enhanced disease surveillance and outbreak control in developing nations. In addition, we hope to promote AIDO as a training tool for epidemiologists. Owing to the breadth of information contained, and the wide array of analytics available, subject matter expert reviewers have mentioned that this is a logical next step in AIDO’s development. We also envision application of AIDO for investigation of syndromic outbreaks through the development of a feature that would allow the identification of a causative agent for syndromic input into AIDO. These analyses would focus on identifying a pathogen within disease families (eg, gastroenteritis mosquito-borne disease family).

### Conclusions

History often repeats itself. This is the simple underlying premise of AIDO. Rather than using limited elements of historical outbreak data to merely inform mathematical models, AIDO takes advantage of the entire story of a historical outbreak to offer examples of similar outbreaks for a current unfolding event. Importantly, it facilitates the use of limited and isolated information in the early stages of an outbreak to make decisions. Information obtained from AIDO can also be used to improve outbreak modeling parameters. AIDO aids in the investigation of disease outbreaks by contextualizing an unfolding outbreak with closely matching historical outbreaks. We posit that by providing diverse layers of information, visuals, and analytics, AIDO furnishes a comprehensive picture that may allow the user to make informed decisions about outbreak control. The tool allows *no cost* epidemiological evaluations, as it is freely available on the internet and can be used iteratively during the early stages of an outbreak. We offer this analytic to the global infectious disease surveillance community as a rapid and facile decision support tool that can be easily accessed—a simple yet useful resource that is the first of its kind.

## Appendix

Multimedia Appendix 1 Disease library details. This table shows all diseases currently included in AIDO and the number of outbreaks included in each disease. The number of countries represented in each library helps to describe the geographic variation represented therein. Properties included in the similarity algorithm for each disease (per the statistical procedure described in the main text) are also given. Some diseases had fewer than 10 outbreaks, which was determined to be too little data to run the statistical analysis. In addition, some other diseases did not have any statistically significant properties. These outbreaks are available to interact with via “browsing”, but do not include the similarity matching algorithm analytics.

Multimedia Appendix 2 Automatic weight calculation algorithm.

Multimedia Appendix 3 Supplementary Table 2A and 2B: Mosquito borne and vaccine preventable disease property comparison. Table 2A compares properties for eight mosquito-borne diseases. Table 2B shows a similar comparison for eight vaccine-preventable diseases. In general, we find that similar diseases may have some properties in common, but properties tend to be distinct across diseases. Note that no statistically significant properties were identified within our mumps library.

Multimedia Appendix 4 Additional case study examples; chikungunya, measles and dengue outbreaks in 2017-2018. AIDO was used to evaluate three recent outbreaks. AIDO input data and comparison of AIDO results and the actual outbreak data is given. Results showed that AIDO was able to provide estimates of case count, duration for the outbreak as well as identify distinctive features with only early stage data used as input.

## Abbreviations

AIDO: Analytics for the Investigation of Disease Outbreaks
ANOVA: analysis of variance
CADE: classifier-adjusted density estimation
CDC: Centers for Disease Control and Prevention
HDI: Human Development Index
PI: prediction interval
