# Supplementary material for: Analytics for Investigation of Disease Outbreaks: Web-Based Analytics Facilitating Situational Awareness in Unfolding Disease Outbreaks
Source: JMIR Public Health Surveill. 2019 Feb 25;5(1):e12032. doi: 10.2196/12032 (PMC6409513; doi:10.2196/12032)
Supplement: Multimedia Appendix 1 [file publichealth_v5i1e12032_app1.docx]

**Supplementary Table 1**

|  | **Disease** | **# Outbreaks** | **# Countries represented** | **Properties in algorithm** |
| --- | --- | --- | --- | --- |
| 1. | Anthrax | 14 | 8 | HDI  Physician density |
| 2. | Brucellosis | 10 | 10 | Disease status  Physician density  HDI |
| 3. | Campylobacteriosis | 17 | 9 | Pathogen source  Contamination source |
| 4. | Chikungunya | 28 | 19 | Precipitation  HDI |
| 5. | Cholera | 31 | 20 | HDI  CFR  Rainy vs. dry |
| 6. | Crimean-Congo Hemorrhagic Fever | 10 | 5 | Physician density  CFR |
| 7. | Dengue | 22 | 16 | Physician density  Climate  Population (discrete) |
| 8. | Ebola | 19 | 10 | Case definition  Physician density |
| 9. | Foot and Mouth Disease | 14 | 13 | No statistically significant properties |
| 10. | Gastroenteritis | 6 | 6 | Library too small to perform analysis |
| 11. | Japanese Encephalitis | 13 | 5 | Population  Rainy vs. dry  Rural vs. urban  Vector type  CFR |
| 12. | Lassa Fever | 3 | 1 | Library too small to perform analysis |
| 13. | Leishmaniasis | 8 | 8 | Library too small to perform analysis |
| 14. | Leptospirosis | 18 | 13 | Rural vs. urban  Rainy vs. dry |
| 15. | Malaria | 25 | 19 | Disease status  Outbreak pathogen |
| 16. | Marburg | 4 | 3 | Library too small to perform analysis |
| 17. | Measles | 35 | 26 | Vaccination status (country)  Vaccination status (region)  Physician density  Climate |
| 18. | Meningococcal Disease | 24 | 14 | Outbreak pathogen  Climate  HDI  Rural vs. urban  Special population group |
| 19. | Middle East Respiratory Syndrome | 4 | 3 | Library too small to perform analysis |
| 20. | Monkeypox | 10 | 6 | Climate  HDI  Physician Density |
| 21. | Mumps | 24 | 14 | No statistically significant properties |
| 22. | Nipah | 7 | 3 | Library too small to perform analysis |
| 23. | Novel Influenza A | 48 | 19 | No statistically significant properties |
| 24. | Norovirus | 33 | 18 | Population (discrete) |
| 25. | Pertussis | 12 | 7 | Population (continuous)  Vaccination status (country)  Physician density  Median age  HDI |
| 26. | Plague | 16 | 12 | Population (continuous)  Pathogen source  CFR  Climate  HDI  Rural vs. urban |
| 27. | Polio | 21 | 16 | HDI  Disease status  Vaccination status (country)  Population movement  Population (continuous) |
| 28. | Porcine Epidemic Diarrhea Virus | 5 | 4 | Library too small to perform analysis |
| 29. | Q Fever | 24 | 15 | Animal contact  HDI  Affected animal  Proximity to animal |
| 30. | Rift Valley Fever | 10 | 8 | HDI  Outbreak curve  Population (continuous) |
| 31. | Rubella | 13 | 12 | Population (discrete)  HDI  Physician density  Median age  Case definition |
| 32. | Salmonellosis | 31 | 13 | Pathogen source  Outbreak pathogen  HDI  Contamination source  Case definition |
| 33. | Severe Acute Respiratory Syndrome (SARS) | 5 | 5 | Library too small to perform analysis |
| 34. | Shiga Toxin-Producing *E. coli* | 19 | 9 | Population (discrete)  Outbreak pathogen  Outbreak curve  HDI  Contamination source |
| 35. | Shigellosis | 18 | 10 | Contamination source  Population (continuous) |
| 36. | Tularemia | 21 | 10 | Case definition |
| 37. | West Nile Virus | 17 | 9 | HDI  Disease status |
| 38. | Yellow Fever | 20 | 8 | Physician density  Transmission mode  Population (discrete)  HDI |
| 39. | Zika | 14 | 9 | Population (discrete) |
|  | **Total** | **673** |  |  |

**Supplementary Table 1: Disease library details.** This table shows all diseases currently included in AIDO and the number of outbreaks included in each disease. The number of countries represented in each library helps to describe the geographic variation represented therein. Properties included in the similarity algorithm for each disease (per the statistical procedure described in the main text) are also given. Some diseases had fewer than 10 outbreaks, which was determined to be too little data to run the statistical analysis. In addition, some other diseases did not have any statistically significant properties. These outbreaks are available to interact with via “browsing”, but do not include the similarity matching algorithm analytics.
