# Supplementary material for: Analytics for Investigation of Disease Outbreaks: Web-Based Analytics Facilitating Situational Awareness in Unfolding Disease Outbreaks
Source: JMIR Public Health Surveill. 2019 Feb 25;5(1):e12032. doi: 10.2196/12032 (PMC6409513; doi:10.2196/12032)
Supplement: Multimedia Appendix 2 [file publichealth_v5i1e12032_app2.docx]

## Appendix 1: Automatic weight calculation algorithm

The process to assign weights for each property involves a modified sensitivity analysis in which the results of Equation 1 (shown in the main body of the manuscript) are compared when each property is left out. Specifically, the changes in outbreak order are recorded when different properties are no longer considered. If removing a property has a large effect on the order of the outbreaks, it can be inferred that the excluded property is important and should be given a greater weight. In order to run this analysis, we use Equation 1 to perform pairwise comparisons between historical outbreaks. Because no weights are known a priori for Equation 1, all weights are initially set to the same value for this process.

For all diseases, the case count and duration properties are given the greatest weight and are not considered in the processing done for the weighting algorithm.

The weighting algorithm first iterates over all outbreaks in the disease library, selecting a different outbreak at each step as the current test outbreak. For each of these test outbreaks, a list of “full scores” is calculated using Equation 1 to compare the test outbreak against every outbreak in the disease library. This list of full scores describes the order that the library outbreaks would have if the user entered the test outbreak's data into AIDO. In addition to calculating the full scores, the algorithm iterates over every selected disease property for each test outbreak to calculate each test outbreak's “partial scores”. To calculate a partial score for each disease property, that property is removed, and Equation 1 is used to calculate the similarity score between the test outbreak and the rest of the library. This allows us to see the effect of removing the given property. Full and partial scores are then compared to see the effect of individual properties on the overall algorithm. From this, a ranking of disease properties can be found which allows the algorithm to assign the weights needed in Equation 1 (see main text).

In order to create this property ranking, two metrics are calculated for each property: the difference value and the similarity value. The difference value for any property describes how many outbreak scores are affected by that property, while the similarity value represents how that property changes the overall ordering of the outbreaks.

In order to calculate the difference value, the full and partial scores are first rounded to two decimal places. The algorithm then iterates over every outbreak in the disease library and, for each test outbreak, counts the number of unique full score and partial score values. The difference value for any property is the average across the outbreaks of the absolute value of the difference between the number of unique full score values and the number of unique partial score values for the given property (see Appendix Table 1).

The similarity value for a property is the number of outbreaks that keep the same position when the outbreaks are ordered based on the full and partial scores. For each partial score, if an outbreak keeps the same position in the search results as it did in the search results produced by the full scores, the similarity value for the current property is incremented by one (see Appendix Table 2).

At this point, a difference and similarity value has been calculated for every property defined for the disease. Using the difference and similarity values, we rank properties such that properties that have a greater difference value are given preference, and properties that have a smaller similarity value are given preference. The final ranking is created by averaging each property's position in the difference and similarity rankings. Using this final ranking, the weight for the $i^{th}$ property is calculated using the following formula:

$w_{i}=\frac{K-r_{i}+1}{\sum_{j=1}^{K} K- r_{j}+1}$ (2)

where $K$ is the number of properties in the disease and $r_{i}$ is the rank of the $i^{th}$ property. This process has been automated so that it can be run every time new data are uploaded to AIDO.

| Test outbreak | Outbreak | Full Score | Partial score 1 | Partial score 2 | Partial score 3 | Partial score 4 |
| --- | --- | --- | --- | --- | --- | --- |
| Outbreak 1 | Outbreak 1 | 1 | 1 | 1 | 1 | 1 |
| Outbreak 1 | Outbreak 2 | 0.62 | 0.83 | 0.67 | 0.67 | 0.67 |
| Outbreak 1 | Outbreak 3 | 0.75 | 0.83 | 0.67 | 0.5 | 0.5 |
| Outbreak 1 | Outbreak 4 | 0.75 | 0.83 | 0.83 | 0.58 | 0.58 |
| Number of unique scores | | 3 | 2 | 3 | 4 | 4 |
| Difference value | |  | 1 | 0 | 1 | 1 |

Appendix Table 1: Example calculation of the difference value for a single outbreak. The “full score” column shows the scores received when all properties are included. The “Partial score” columns show the scores received when each property is excluded. The “Number of unique scores” row shows the count of the unique scores in that column, while the “Difference value” row shows the absolute value of the difference between number of unique scores for each partial score and the full score. A similar table is calculated for each outbreak, and the final difference value for each property is calculated by averaging the difference values that were calculated for each outbreak.

| Test outbreak | Full score order | Partial score 1 Order | Partial score 2 Order | Partial score 3 Order | Partial score 4 Order |
| --- | --- | --- | --- | --- | --- |
| Outbreak 1 | Outbreak 1 | Outbreak 1 | Outbreak 1 | Outbreak 1 | Outbreak 1 |
| Outbreak 1 | Outbreak 3 | Outbreak 2 | Outbreak 4 | Outbreak 2 | Outbreak 2 |
| Outbreak 1 | Outbreak 4 | Outbreak 3 | Outbreak 2 | Outbreak 4 | Outbreak 4 |
| Outbreak 1 | Outbreak 2 | Outbreak 4 | Outbreak 3 | Outbreak 3 | Outbreak 3 |
| Count | | 1 | 1 | 2 | 2 |

Appendix Table 2: Example calculation of the similarity value calculation. Each column shows how the outbreaks are ordered based on the scores they received. The “Full score order” column shows the ordering when all properties are included, while the “Partial score order” columns show the ordering when each of the properties are removed. The shaded cells are the outbreaks that count toward the similarity value. These shaded cells are counted for each column and added to the “Count” row, which represents the similarity value for each property for this particular test outbreak. A similar table is calculated for every outbreak. The similarity values for each property are averaged to find the final similarity value for the given property
