# Supplementary material for: Analytics for Investigation of Disease Outbreaks: Web-Based Analytics Facilitating Situational Awareness in Unfolding Disease Outbreaks
Source: JMIR Public Health Surveill. 2019 Feb 25;5(1):e12032. doi: 10.2196/12032 (PMC6409513; doi:10.2196/12032)
Supplement: Multimedia Appendix 3 [file publichealth_v5i1e12032_app3.docx]

**Supplementary Table 2A and 2B**

| **chikungunya** | **dengue** | **WNV** | **malaria** | **JEV** | **zika** | **RVF** | **YF** |
| --- | --- | --- | --- | --- | --- | --- | --- |
| Precipitation |  |  |  |  |  |  |  |
|  | Physician density |  |  |  |  |  | Physician density |
| HDI |  | HDI |  |  |  | HDI | HDI |
|  | Climate |  |  |  |  |  |  |
|  | Population group |  |  | Population | Population group | Population group | Population group |
|  |  | Disease status | Disease status |  |  |  |  |
|  |  |  | Outbreak pathogen |  |  |  |  |
|  |  |  |  | Rural or urban |  |  |  |
|  |  |  |  | Vector genus and species |  |  |  |
|  |  |  |  | Case fatality rate (cfr) |  | Case fatality rate (cfr) |  |
|  |  |  |  | Attack rate |  |  |  |
|  |  |  |  |  |  | Epidemic curve shape |  |
|  |  |  |  |  |  |  | Transmission mode |
|  |  |  |  | Rainy or dry period |  |  |  |

| **measles** | **mumps** | **rubella** | **pertussis** | **polio** | **meningococcal** | **yellow fever** | **JEV** |
| --- | --- | --- | --- | --- | --- | --- | --- |
| Vaccination percentage of the country |  |  | Vaccination percentage of the country | Vaccination percentage of the country |  |  |  |
| Vaccination percentage of the affected region |  |  |  |  |  |  |  |
| Physician density |  | Physician density | Physician density |  |  | Physician density |  |
| climate |  |  |  |  | Climate |  |  |
|  |  |  | Population | Population |  | Population group | Population |
|  |  | Median age | Median age |  |  |  |  |
|  |  | HDI | HDI | HDI | HDI | HDI |  |
|  |  |  |  | Disease endemicity status |  |  |  |
|  |  |  |  | Population movement |  |  |  |
|  |  |  |  |  | Outbreak pathogen |  |  |
|  |  |  |  |  | Rural or urban |  | Rural or urban |
|  |  |  |  |  | Special population group |  |  |
|  |  |  |  |  |  | Transmission mode |  |
|  |  |  |  |  |  |  | Vector genus and species |
|  |  |  |  |  |  |  | Case fatality rate (cfr) |
|  |  |  |  |  |  |  | Attack rate |
|  |  |  |  |  |  |  | Rainy or dry period |

**Supplementary Table 2A and 2B: Mosquito borne and vaccine preventable disease property comparison.** Table 2A compares properties for eight mosquito-borne diseases. Table 2B shows a similar comparison for eight vaccine-preventable diseases. In general, we find that similar diseases may have some properties in common, but properties tend to be distinct across diseases. Note that no statistically significant properties were identified within our mumps library.
