# Supplementary material for: Analytics for Investigation of Disease Outbreaks: Web-Based Analytics Facilitating Situational Awareness in Unfolding Disease Outbreaks
Source: JMIR Public Health Surveill. 2019 Feb 25;5(1):e12032. doi: 10.2196/12032 (PMC6409513; doi:10.2196/12032)
Supplement: Multimedia Appendix 4 [file publichealth_v5i1e12032_app4.docx]

**Supplementary Table 3**

| **Case study** | **Input for case count and duration** | **Input for disease specific properties** | **Top 3 matching historical outbreaks and similarity score** | **Similar features between top matching outbreaks and case study** |
| --- | --- | --- | --- | --- |
| **Chikungunya outbreak in Dhaka, Bangladesh**  (6000-7000 cases of Chikungunya were reported from Bangladesh during April-August, 2017 and the outbreak involved multiple waves of peak cases) | 100 cases in 30 days | Precipitation – winter dry  HDI – 0.576 | India 2006 (95%)  Cambodia 2012 (89%)  Thailand 2008 (83%) | Estimated total case count in 5000-10,000 range  Estimated duration 4-5 months  Multiple waves of peak cases possible |
| **Measles outbreak in Taiwan and Japan** (About 100 cases of measles have been reported from Taiwan and Japan in this 2018 outbreak) | 22 cases in 24 days | Vaccination % of country – 99%  Vaccination % of region - 99%  Physician density – 1.812  Climate–warm temperature (C ) | France 2008 (87%)  Italy 2013 (87%)  United Kingdom 2012 (86%) | Estimated case count 150-400 cases in first three months  Spread to neighboring countries  Travel related and nosocomial transmission |
| **Dengue outbreak in Kerala, India** (19,000 dengue cases were reported during the 2017 monsoon season in Kerala and cases are being reported in 2018) | 680 cases in 18 days | Physician density – 0.758  Climate – equatorial  Population group – 30-300 million | Singapore 2012 (77%)  Singapore 2005 (76%)  Dominican republic (73%) | Estimated total case count 15,000-20,000 possible in year one  Possible second wave of cases during the next rainy season and/or during 2018 monsoon season |

**Supplementary Table 3: Additional case study examples; chikungunya, measles and dengue outbreaks in 2017-2018.** AIDO was used to evaluate three recent outbreaks. AIDO input data and comparison of AIDO results and the actual outbreak data is given. Results showed that AIDO was able to provide estimates of case count, duration for the outbreak as well as identify distinctive features with only early stage data used as input.
